# Oral Tori in Chronic Hemodialysis Patients

**DOI:** 10.1155/2015/897674

**Published:** 2015-03-31

**Authors:** Pei-Jung Chao, Huang-Yu Yang, Wen-Hung Huang, Cheng-Hao Weng, I-Kuan Wang, Aileen I. Tsai, Tzung-Hai Yen

**Affiliations:** ^1^Department of Pediatric Dentistry, Chang Gung Memorial Hospital and College of Medicine, Chang Gung University, Linkou 333, Taiwan; ^2^Department of Nephrology and Division of Clinical Toxicology, Chang Gung Memorial Hospital and College of Medicine, Chang Gung University, Linkou 333, Taiwan; ^3^Kidney Research Center, Chang Gung Memorial Hospital, Linkou 333, Taiwan; ^4^Department of Nephrology, China Medical University Hospital and College of Medicine, China Medical University, Taichung 404, Taiwan; ^5^Center for Tissue Engineering, Chang Gung Memorial Hospital, Linkou 333, Taiwan

## Abstract

*Background.* This study investigated the epidemiology of torus palatinus (TP) and torus mandibularis (TM) in hemodialysis patients and analyzed the influences of hyperparathyroidism on the formation of oral tori.* Method*. During 2013, 119 hemodialysis patients were recruited for dental examinations for this study.* Results*. The prevalence of oral tori in our sample group was high at 33.6% (40 of 119). The most common location of tori was TP (70.0%), followed by TM (20.0%), and then both TP and TM (10.0%). Of the 40 tori cases, most (67.5%) were <2 cm in size; moreover, the majority (52.5%) were flat in shape. In symmetry, most (70.0%) occurred in the midline, followed by bilateral sides (20.0%). Notably, the levels of intact parathyroid hormone did not differ in patients with or without tori (*P* = 0.611). Furthermore, patients with tori did not differ from patients without tori in inflammatory variables such as log high-sensitivity C-reactive protein (*P* = 1.000) or nutritional variables such as albumin (*P* = 0.247). Finally, there were no differences between patients with and without tori in adequacy of dialysis (*P* = 0.577).* Conclusions*. Neither hyperparathyroidism nor inflammation malnutrition syndrome was found to contribute to the formation of oral tori in chronic hemodialysis patients. Further studies are warranted.

## 1. Introduction

Tori or exostoses are described as nonpathologic, localized bony protuberances that arise from the cortical bone and sometimes the spongy layer. The two most common exostoses that occur in two specific intraoral locations, on the midline of the hard palate and the lingual aspect of the mandible in the cuspid/premolar region, are termed TP and TM [1]. TP is an exophytic nodular mass of bone that rises along the midline suture of the hard palate. In contrast, TM is a bony exophytic growth located on the cuspid/premolar area of the lingual surface of the mandible and superior, usually bilaterally, to the mylohyoid ridge [1]. Morphologically, tori are classified as flat, spindle, nodular, and lobular [1].

The discovery of these exostoses usually occurs incidentally during a routine dental examination, as they generally do not produce any symptoms (except in cases of significant growth or in edentulous patients, in which case they can hinder the construction of the prosthesis) [2]. Despite the numerous studies, their origin is unclear [3]; numerous potential causes are presented in literature, but none are definitive. Certain prevalence with respect to ethnic groups, sex, and age has also been observed [4–18].

The exact etiology of oral tori has eluded investigators for decades, but it is believed that the trait is expressed when a certain threshold of genetic and local environmental factors is surpassed [19–21]. Historically, studies on the etiology of these bony lesions have focused on genetic and environmental influences, but they have neglected to investigate the broad scope of interdependent factors involved in bone or mineral metabolism.

Taiwan continues to report the highest rate of prevalent end-stage renal disease (ESRD) in the world. According to 2014 Annual Data Report of United States Renal Data System [22], the number of ESRD patients per million receiving chronic dialysis in 2012 varied more than 20-fold across countries, from 2,902 and 2,365 in Taiwan and Japan, respectively, to 133–185 in South Africa, Russia, and the Philippines [22]. Therefore, it is speculated that many of our hemodialysis patients might have a different epidemiology of oral tori due to an underlying chronic kidney disease-mineral and bone disease or inflammation malnutrition syndrome.

Abnormal calcium, phosphorus, and vitamin D metabolism are very common in patients with ESRD [23]. Metabolic disturbances in these patients result in the prolonged stimulation of the parathyroid glands. This results in the increased synthesis and release of parathyroid hormone and causes secondary hyperparathyroidism. Hyperparathyroidism causes the skeletal disturbances that are characteristic of renal osteodystrophy [23].

In a pilot study, Sisman et al. [16] investigated the prevalence, size, location, and shape of TP in 91 ESRD patients receiving peritoneal dialysis. A higher prevalence of TP (41.7%) and the significant relationship between duration of renal dialysis and size of TP were reported. They attributed the development of TP to an underlying disorder, such as renal osteodystrophy [16].

Therefore, the objective of this study was to undertake a broader assessment of potential environmental influences and, in doing so, address the following question: in hemodialysis patients with oral tori, are there associations with molar relationships, medical conditions, chronic kidney disease-mineral and bone disease, or inflammation malnutrition syndrome?

## 2. Material and Methods

### 2.1. Ethical Statement

This clinical study followed the Declaration of Helsinki and was approved by the Medical Ethics Committee of Chang Gung Memorial Hospital.

### 2.2. Patients

All hemodialysis patients were recruited from Chang Gung Memorial Hospital at Linkou, Taiwan. This observational study included 119 patients. All patients who agreed to participate in this study were enrolled, excluding those with malignancies [24], active infectious diseases, hospitalizations, or surgery or kidney transplants in the past 3 months and those on hemodialysis for less than 3 months or intoxicated by lead [25, 26] or cadmium [27, 28]. All enrolled patients underwent 4 hours of hemodialysis, 3 times a week. Hemodialysis was performed with single-use hollow-fiber dialyzers equipped with modified cellulose-based polyamide or polysulfone membranes. The dialysate used was a standard ionic composition and bicarbonate-based buffer. Patients with hypertension took antihypertensive medications regularly, except diuretics. Blood sugar was controlled with insulin therapy and the glucose level regularly monitored. Smoking habits and alcohol consumption were also recorded.

### 2.3. Groups

Patients who met the inclusion criteria were classified into 2 groups according to the presence or absence of oral tori.

### 2.4. Laboratories

Blood specimens were collected within a few days of a clinical examination that occurred during stable hemodialysis sessions to minimize the effect of any acute events. Blood was drawn from the arterial end of the vascular access immediately before hemodialysis, centrifuged, and then stored at −70°C until used in assays. Serum levels of albumin, blood urea nitrogen, and creatinine, transferring saturation, and normalized protein catabolic rate were measured and used as nutritional markers. The high-sensitivity C-reactive protein, which was used as an inflammatory biomarker, was analyzed by immunonephelometry (Nanopia CRP; Daiichi, Tokyo, Japan). The lowest detection limit was 0.15 mg/L. All other data were obtained with standard laboratory procedures using an automatic analyzer. The normalized protein catabolic rate in hemodialysis patients was calculated using validated equations, and it was normalized to body weight. Dialysis clearance of urea was expressed as *Kt*/*V*_urea_, as reported by Daugirdas [29]. Serum levels of calcium, phosphate, and intact parathyroid hormone were also measured and the corrected serum calcium level was calculated as follows: calcium (mg/dL) = [0.8 (4.0 − albumin [g/dL])].

### 2.5. Diagnosis of Oral Tori

**Figure 1:**
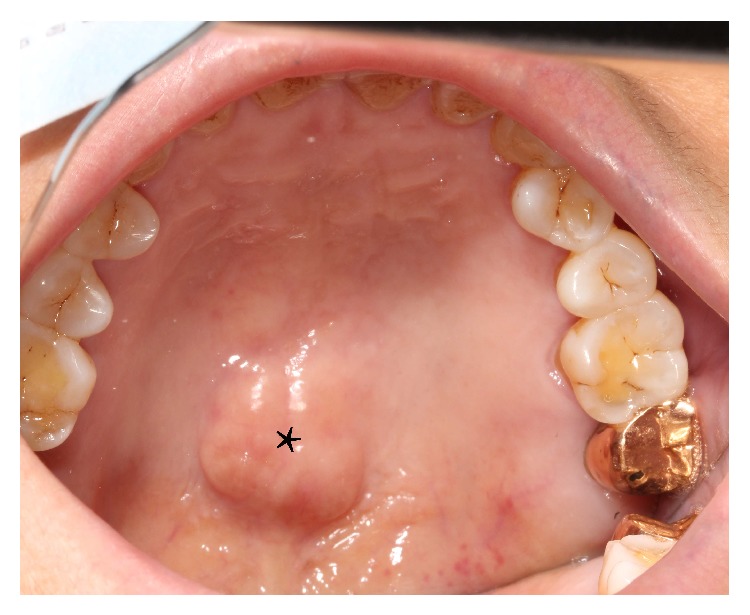
Torus palatinus. Intraoral view of one of the hemodialysis patients with a flat torus palatinus (asterisk) which is an exophytic nodular bony mass that arises along the midline of the hard palate.

The same dentist examined all patients and used mouth mirrors or tongue blades to check the oral condition of these patients. The examination for oral tori consisted of inspection and palpation. TP (Figure 1) was defined as a raised bony exostosis along the midline of the hard palate whereas TM was defined as exostosis that develops along the lingual aspect of the mandible. The maximum elevation of the outgrowth of tori was used to measure the size of tori. Tori were graded according to previous description as being >2 cm or <2 cm using a periodontal probe, as described by Gorsky et al. [4]. The shape of tori was classified as flat, spindle, nodular, or lobular according to the criteria described by Jainkittivong et al. [5]. The locations of tori were classified as being in the upper arch, lower arch, or upper and lower arches. The molar relationship was classified as Class I, Class II, or Class III, as defined previously [30].

### 2.6. Statistical Analysis

Continuous variables were expressed as a mean with a standard deviation, while categorical variables were expressed as numbers and percentages in brackets. All data were tested for normality of distribution and equality of standard deviation before analysis. As the high-sensitivity C-reactive protein data were not normally distributed, these data were log transformed before being entered into the regression model. Comparisons between the 2 groups of patients were performed using Student's *t*-test for quantitative variables and Chi-square or Fisher's exact tests for categorical variables. The criterion for significance was a 95% confidence interval to reject the null hypothesis. All analyses were performed using IBM SPSS Statistics Version 20.0.

## 3. Results

### 3.1. Subject Characteristics

The patients were aged 59.8 ± 14.7 years with roughly equal sex distribution (Table 1). Of the 119 patients with ESRD, 40 were found to have oral tori, and the prevalence rate in our hemodialysis population was 33.6%. Nevertheless, there were no significant differences in baseline demographic variables between both groups (*P* > 0.05).

### 3.2. Laboratory Findings

Patients with oral tori did not differ from patients without tori in their level of intact parathyroid hormone (354.0 ± 477.3 pg/mL versus 392.7 ± 340.3 pg/mL, *P* = 0.611, Table 2). Furthermore, patients with and without oral tori did not differ in inflammatory variables such as log high-sensitivity C-reactive protein (0.6 ± 0.6 mg/L versus 0.6 ± 0.6 mg/L, *P* = 1.000) or nutritional variables such as albumin (4.0 ± 0.3 g/dL versus 3.9 ± 0.1 g/dL, *P* = 0.247).

### 3.3. Dialysis-Related Data

There were no significant differences in hemodialysis adequacy between patients with and without oral tori (*Kt*/*V*, 1.7 ± 0.4 versus 1.7 ± 0.3, *P* = 0.577, Table 3).

### 3.4. Clinical Findings of Oral Tori

As shown in Table 4, the most common location of tori was TP (70.0%), followed by TM (20.0%). The incidence of tori that occurred in both upper and lower arch was 10.0%. Of the 40 tori cases, most (67.5%) were <2 cm in size and, in addition, most (52.5%) were flat in shape. In symmetry, most (70.0%) were in the midline, followed by bilateral sides (20.0%).

### 3.5. Molar Relationship

The molar relationship could not be defined in most patients (50.4%) due to the loss of first molars (Table 5). There was no significant difference in molar relationship between both groups (*P* = 0.400).

## 4. Discussion

Few data are available regarding the prevalence rate of oral tori in chronic hemodialysis patients; this is the first study examining the prevalence of oral tori in patients with ESRD in Taiwan. Our data revealed that 40 out of 119 (33.6%) hemodialysis patients had oral tori. TP generally occurs in anywhere from 4.1 to 60.5% of the population, and different studies have reported marked differences among various ethnic groups (Table 6). Chiang et al. [17] investigated oral mucosal anomalies in 2050 dental patients of a hospital in Taiwan and reported that the prevalence rate of TP was 21.1%. Thus, this study showed the slightly higher prevalence of TP (23.5%) in patients receiving hemodialysis. Our results also demonstrated a high prevalence of TP, similar to the results of a study by Sisman et al. [16]; 41.7% of the patients had TP.

The present study examined not only TP but also TM. Sisman et al. [16] only reported the prevalence of TP. Our study showed that TP occurred in 28 patients (23.5%), TM occurred in 8 patients (6.7%), and both TP and TM occurred in 4 patients (3.4%). It appears that TP is the major form of oral tori in patients undergoing hemodialysis therapy. However, Chiang et al. [17] found that the prevalence of TM (24.2%) was slightly higher than the prevalence of TP (21.1%).

The TP shapes were classified as flat, spindle, nodular, or lobular according to the criteria described by Jainkittivong et al. [5]. However, the TM shapes were not classified. In this study, we found flat tori to be the most common TP type. Numerous studies agree with this finding of flat TP being the most common shape [5, 31–33]. Studies by Reichart et al. [6], Sisman et al. [16], and Jainkittivong et al. [5], however, reported that spindle-shaped TP was the most common type. Sisman et al. [7], in 2008, surveyed a regional population in Turkey and reported that flat TP was most common there. Sisman et al. [16] speculated that the difference between these two studies in most common TP type, which were conducted in the same region, might be due to an underlying disorder, such as renal osteodystrophty, in the ESRD patients.

Nevertheless, we found in this study that patients with and without oral tori did not differ in levels of intact parathyroid hormone (*P* = 0.611). Furthermore, patients with and without oral tori also did not differ in inflammatory variables such as log high-sensitivity C-reactive protein (*P* = 1.000) or nutritional variables such as albumin (*P* = 0.247). Therefore, it was very difficult to attribute tori formation in the hemodialysis patients to uremic milieu, inflammation malnutrition syndrome, or renal osteodystrophy. Sisman et al. [16] revealed that the prevalence of TP in ESRD patients undergoing peritoneal dialysis was higher (41.7%) compared to other Turkish reports and especially compared to the study by Sisman et al. [7] (4.1%) that was performed in the same region but in the general population. On the other hand, the present study demonstrated a slightly higher prevalence of TP (23.5%) in hemodialysis patients than general population (21.1%) [17].

The molar relationship could not be defined in most patients (50.4%) due to loss of first molars. In addition, there was no significant difference in molar relationship between patients with and without tori (*P* = 0.400). In 1999, Sonnier et al. [34] examined the prevalence of 3 types of exostoses in a sample of 328 modern American skulls drawn from the collection at the American Museum of Natural History. It was revealed that TP was observed in 56% of all skulls, was commonly associated with second and third molars, and was usually directly lateral to and a mean of 11.4 mm from the greater palatine foramen [34]. Mishra et al. [18] also found that TP was often located at the combined premolar to molar areas. Gorsky et al. [4] reported that the prevalence of TP in the combined molar-premolar area increased with age, whereas in the molar area it decreased, expressing a significant relation between location and age (*P* < 0.01). On the other hand, Sawair et al. [11] demonstrated that TM was mostly located at the premolar region (65.4%).

Table 6 compares the prevalence of oral tori from different studies. It was revealed that the prevalence of TP ranged from 4.1 to 60.5% [4–18] and the prevalence seemed to vary from country to country. Although the epidemiology of oral tori has been studied comprehensively in literature, there is only one group [16] reporting a high prevalence rate of TP (41.7%) in the peritoneal dialysis population. Nevertheless, the comparison of prevalence rates between patients with and without renal dialysis was not accurate without an appropriate age and gender adjusted control group.

## 5. Conclusion

In conclusion, neither hyperparathyroidism nor inflammation malnutrition syndrome was found to contribute to the formation of oral tori in our patients. Nevertheless, the current study is limited by a small sample size, short follow-up duration, and lack of histopathology analysis between spontaneous and hemodialysis-induced tori. Further studies are warranted.

## Figures and Tables

**Table 1:** Baseline characteristics of hemodialysis patients with or without oral tori formation (*n* = 119).

Variable	All patients (*n* = 119)	Patients with oral tori (*n* = 40)	Patients without oral tori (*n* = 79)	*P* value
Age, year	59.8 ± 14.7	60.0 ± 13.2	59.7 ± 15.5	0.905
Male sex, *n* (%)	60 (50.4)	18 (45.0)	42 (53.2)	0.400
Body mass index, kg/m^2^	22.3 ± 3.4	22.7 ± 3.7	22.1 ± 3.2	0.360
Diabetes mellitus, *n* (%)	47 (39.5)	19 (47.5)	28 (35.4)	0.204
Hypertension, *n* (%)	66 (55.5)	23 (57.5)	43 (54.4)	0.750
Smoking habit, *n* (%)	27 (22.7)	8 (20)	19 (24.1)	0.618
Alcohol consumption, *n* (%)	20 (16.8)	5 (12.5)	15 (19.0)	0.371

**Table 2:** Laboratory findings of hemodialysis patients with or without oral tori formation (*n* = 119).

Variable	All patients (*n* = 119)	Patients with oral tori (*n* = 40)	Patients without oral tori (*n* = 79)	*P*
Blood urea nitrogen, mg/dL	73.4 ± 20.3	73.5 ± 14.6	73.4 ± 22.7	0.976
Creatinine, mg/dL	10.3 ± 2.2	10.1 ± 2.3	10.5 ± 2.2	0.370
Uric acid, mg/dL	7.1 ± 1.3	6.8 ± 1.2	7.2 ± 1.3	0.117
Sodium, mEq/L	137.9 ± 3.1	137.8 ± 3.3	138.2 ± 2.7	0.521
Potassium, mEq/L	4.8 ± 0.8	4.8 ± 0.8	4.8 ± 0.7	0.767
Chloride, mEq/L	97.8 ± 3.5	97.8 ± 3.2	97.9 ± 3.7	0.916
Calcium, mg/dL	9.6 ± 1.0	9.6 ± 0.9	9.7 ± 1.0	0.571
Inorganic phosphorus, mg/dL	5.3 ± 1.6	5.1 ± 1.3	5.4 ± 1.7	0.346
Bicarbonate, mmol/L	23.0 ± 2.7	23.0 ± 3.1	23.0 ± 2.5	0.942
Fasting glucose, mg/dL	126.4 ± 69.9	125.4 ± 52.9	126.9 ± 77.4	0.912
Albumin, g/dL	3.9 ± 0.3	4.0 ± 0.3	3.9 ± 0.1	0.247
Total bilirubin, mg/dL	0.3 ± 0.2	0.3 ± 0.1	0.3 ± 0.2	0.547
Aspartate aminotransferase, U/L	20.6 ± 9.1	19.3 ± 6.3	21.3 ± 10.1	0.241
Alanine aminotransferase, U/L	16.6 ± 12.0	17.5 ± 15.4	16.1 ± 10.0	0.544
Alkaline phosphatase, U/L	95.7 ± 86.1	96.9 ± 94.0	95.1 ± 82.5	0.917
Gamma-glutamyl transferase, U/L	42.8 ± 62.6	41.9 ± 51.6	43.2 ± 67.8	0.918
Total cholesterol, mg/dL	168.7 ± 42.1	173.5 ± 29.2	166.2 ± 47.3	0.379
High-density lipoprotein, mg/dL	44.0 ± 17.7	42.2 ± 15.5	44.9 ± 18.7	0.434
Low-density lipoprotein, mg/dL	95.1 ± 35.9	99.2 ± 27.9	93.0 ± 39.3	0.383
Triglyceride, mg/dL	149.9 ± 99.4	162.5 ± 87.1	143.4 ± 105.0	0.325
Red blood cell count, 10^6^/uL	3.5 ± 0.6	3.6 ± 0.6	3.4 ± 0.5	0.038^*^
Hemoglobin, g/dL	10.2 ± 1.4	10.5 ± 1.3	10.0 ± 1.3	0.058
Hematocrit, %	31.2 ± 4.1	32.2 ± 4.0	30.6 ± 4.0	0.048^*^
Mean corpuscular volume, fL	91.0 ± 6.6	90.2 ± 7.3	91.3 ± 6.2	0.367
Red blood cell distribution width, %	14.4 ± 1.4	14.2 ± 1.2	14.5 ± 1.4	0.230
Platelet count, 10^3^/uL	190.8 ± 58.7	199.7 ± 61.1	186.2 ± 57.3	0.239
White blood cell count, 10^3^/uL	6.5 ± 1.9	7.1 ± 2.0	6.2 ± 1.9	0.025^*^
Intact parathyroid hormone, pg/mL	379.7 ± 390.1	354.0 ± 477.3	392.7 ± 340.3	0.611
Iron, ug/dL	63.9 ± 26.6	69.8 ± 29.0	60.9 ± 25.0	0.086
Total iron binding capacity, ug/dL	251.6 ± 52.0	253.2 ± 55.9	250.8 ± 50.3	0.816
Transferrin saturation, %	26.1 ± 11.5	28.2 ± 11.9	25.0 ± 11.2	0.153
Ferritin, ng/mL	361.3 ± 297.2	284.3 ± 263.7	400.8 ± 307.2	0.043^*^
Log high-sensitivity C-reactive protein, mg/L	0.6 ± 0.6	0.6 ± 0.6	0.6 ± 0.6	1.000

Note: ^*^ *P* < 0.05.

**Table 3:** Dialysis-related data of hemodialysis patients with or without torus formation (*n* = 119).

Variable	All patients (*n* = 119)	Patients with oral tori (*n* = 40)	Patients without oral tori (*n* = 79)	*P* value
Residual glomerular filtration rate, mL/min	2.8 ± 3.25	3.1 ± 3.5	2.7 ± 3.2	0.569
Urea reduction ratio	0.8 ± 0.1	0.8 ± 0.1	0.8 ± 0.1	1.000
Kt/V (Daugirdas [29])	1.7 ± 0.3	1.7 ± 0.4	1.7 ± 0.3	0.577
Normalized protein catabolic rate, g/kg/day	1.4 ± 0.6	1.4 ± 0.7	1.3 ± 0.5	0.460
Time-averaged concentration of urea, mg/dL	45.2 ± 13.9	44.6 ± 9.7	45.5 ± 15.6	0.742

**Table 4:** Clinical findings of oral tori (*n* = 40).

Variable	Findings, *n* (%)				
Location					
Upper	28 (70.0)				
Lower	8 (20.0)	Unilateral	2 (5.0)	Left	1 (2.5)
				Right	1 (2.5)
		Bilateral	6 (15.0)		
Upper and lower	4 (10.0)	Unilateral	2 (5.0)	Left	1 (2.5)
				Right	1 (2.5)
		Bilateral	2 (5.0)		
Size					
<2 cm	27 (67.5)				
>2 cm	13 (32.5)				
Shape					
Flat	21 (52.5)				
Spindle	1 (2.5)				
Nodular	16 (40.0)				
Lobular	2 (5.0)				
Symmetry					
Unilateral	4 (10.0)				
Bilateral	8 (20.0)				
Midline	28 (70.0)				

**Table 5:** Comparison of molar relationship between hemodialysis patients with and without oral tori formation (*n* = 119).

Variable	All patients (*n* = 119)	Patients with torus (*n* = 40)	Patients without torus (*n* = 79)	*P* value
Molar relationship				
No, *n* (%)	60 (50.4)	21 (52.5)	39 (49.4)	0.400
Class I, *n* (%)	50 (42.0)	14 (35.0)	36 (45.6)	
Class II, *n* (%)	3 (2.5)	2 (5)	1 (1.3)	
Class III, *n* (%)	6 (5.0)	3 (7.5)	3 (3.8)	

**Table 6:** Comparison of prevalence rate of oral tori from different studies.

Study	Year	Geographic area	Sample size, *n*	Population	TP, %	TM, %
Reichart et al. [6]	1988	German	1317	Nonuremic	13.5	5.2
Reichart et al. [6]	1988	Thailand	947	Nonuremic	23.1	9.2
Shah et al. [8]	1992	India	1000	Nonuremic	9.5	1.4
Gorsky et al. [4]	1996	Israel	1002	Nonuremic	21.0	
Ruprecht et al. [15]	2000	USA	1600	Nonuremic		16.9^*^
Bruce et al. [9]	2004	Ghana	926	Nonuremic	4.3	12.1
Yildiz et al. [10]	2005	Turkey	1943	Nonuremic	30.9	
Jainkittivong et al. [5]	2007	Thailand	1520	Nonuremic	60.5	32.2
Sawair et al. [11]	2009	Jordan	618	Nonuremic	15.4	25.7
Sisman et al. [7]	2008	Turkey	2660	Nonuremic	4.1	
Yoshinaka et al. [12]	2010	Japan	664	Nonuremic	17	
Simunkovic et al. [13]	2011	Croatia	1679	Nonuremic	42.9	12.6
Mishra et al. [18]	2011	Malaysia	65	Nonuremic	50.8	4.6
Sisman et al. [16]	2012	Turkey	91	Uremic	41.7	
Chiang et al. [17]	2014	Taiwan	2050	Nonuremic	21.1	24.2
Yoshinaka et al. [14]	2014	Japan	664	Nonuremic		29.7
Current study	2014	Taiwan	119	Uremic	23.5	6.7

Note: TP: torus palatinus; TM: torus mandibularis; ^*^radiographic study.
